# Tonsillar Hypertrophy in Chronic Lymphocytic Leukemia and Small Lymphocytic Lymphoma: A Mini-Review

**DOI:** 10.7759/cureus.96963

**Published:** 2025-11-16

**Authors:** Rafal Al-Shibly, Elrazi A Ali, Nabeel M Qasem, Abdulrahman Al-Abdulmalek, Khalil AL-Farsi, Salem AlShemmari, Mohamed A Yassin

**Affiliations:** 1 Internal Medicine, Hamad Medical Corporation, Doha, QAT; 2 Internal Medicine, One Brooklyn Health, Interfaith Medical Center, Brooklyn, USA; 3 Hematology and Medical Oncology, McGill University, Montreal, CAN; 4 Hematology, Sultan Qaboos University Hospital, Muscat, OMN; 5 Hematology, Kuwait Cancer Control Center, Kuwait, KWT; 6 Hematology and Oncology, Hamad General Hospital, Doha, QAT

**Keywords:** airway obstruction, chronic lymphocytic leukemia (cll), obstructive sleep apnea, small lymphocytic lymphoma (sll), tonsillar enlargement

## Abstract

Chronic lymphocytic leukemia (CLL) is a prevalent adult B-cell malignancy that primarily affects individuals over the age of 65. It is characterized by the accumulation of mature B lymphocytes in the blood and bone marrow, whereas small lymphocytic lymphoma (SLL) primarily involves the lymph nodes and lymphoid tissues, with minimal lymphocytosis in the blood. Both CLL and SLL are recognized as a single entity according to iwCLL guidelines. Tonsillar hypertrophy is a rare presentation of CLL and often an overlooked complication. We searched PubMed, Scopus, Semantic Scholar, and Google Scholar up to November 8, 2025, for patients with CLL or SLL and tonsillar enlargement. We identified 14 published cases of CLL/SLL with tonsillar enlargement. The review aims to highlight clinical features, diagnostic challenges, and outcomes. Most patients presented with dysphagia, airway obstruction, or obstructive sleep apnea. Diagnosis frequently required tonsillectomy, followed by histopathology, flow cytometry, and fluorescence in situ hybridization. Management strategies included observation, tonsillectomy for symptom relief and diagnosis, systemic chemoimmunotherapy, or targeted agents such as BTK inhibitors. Most patients achieved remission or symptom improvement; however, a few cases resulted in death due to rapidly progressive disease. This review highlights the importance of considering CLL in adults with unexplained tonsillar hypertrophy. Prompt biopsy, preferably with tonsillectomy and multidisciplinary evaluation, is essential to distinguish this rare entity from other conditions and to guide timely treatment.

## Introduction and background

Chronic lymphocytic leukemia (CLL) is a clonal malignancy of mature B lymphocytes, most commonly diagnosed in elderly patients, and more prevalent in men than in women [1]. While most patients with CLL and small lymphocytic lymphoma (SLL) are asymptomatic, approximately 5-10% present with “B symptoms,” including weight loss, night sweats, fever, fatigue, and lymph node enlargement [1,2]. Lymphadenopathy typically involves the cervical, supraclavicular, and axillary regions, whereas tonsillar enlargement as a primary or early manifestation is uncommon. Unlike bacterial or viral tonsillitis, CLL-related tonsillar hypertrophy is usually painless and afebrile, but it can result in dysphagia, airway obstruction, or obstructive sleep apnea (OSA). Asymmetrical or bilateral tonsillar enlargement in adults should therefore raise suspicion for lymphoproliferative disorders such as CLL. Tonsillar involvement in CLL can mimic benign conditions or other malignancies, often leading to delayed diagnosis and treatment. Historically, CLL has been treated with chemoimmunotherapy regimens, such as fludarabine combined with the anti-CD20 monoclonal antibody rituximab. However, therapeutic strategies have shifted in recent years with the introduction of novel targeted agents, particularly BCL2 inhibitors and Bruton tyrosine kinase (BTK) inhibitors [3]. CLL can involve any lymphoid tissue in the body, such as the spleen, liver, and, since tonsils are part of the reticuloendothelial system, it is expected that tonsils are commonly involved. However, the frequency of tonsillar involvement in patients with CLL and its impact on outcomes is unclear. This mini-review aims to provide an overview of all reported cases of CLL with tonsillar enlargement, highlighting the various clinical manifestations and outcomes, and summarizing the cytogenetic and histopathological features of this rare presentation.

## Review

Method

**Figure 1:**
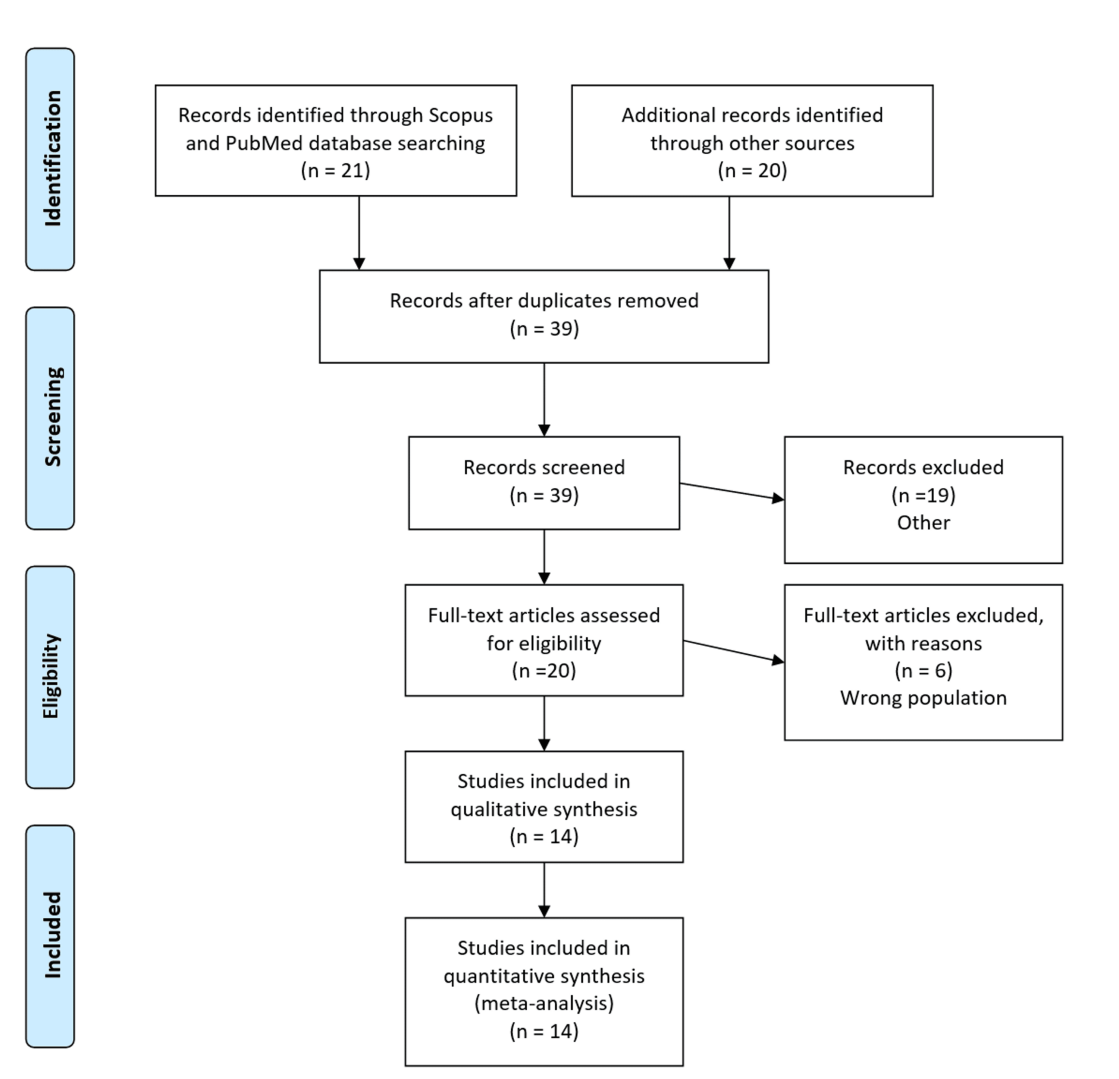
The PRISMA flow diagram detailing the cases of chronic lymphocytic leukemia and small lymphocytic lymphoma that had tonsillar enlargement PRISMA: Preferred Reporting Items for Systematic Reviews and Meta-Analyses

A comprehensive literature search was conducted in PubMed, Scopus, Semantic Scholar, and Google Scholar (Figure 1), using the MeSH terms “chronic lymphocytic leukemia” tonsillar neoplasms. The search strategy used a combination of chronic lymphocytic leukemia OR “small lymphocytic lymphoma”, AND “Tonsillar Enlargement” OR “Tonsilar hypertophy”. The search included all articles published up to November 8, 2025. Inclusion criteria were adults (≥18 years) with chronic lymphocytic leukemia and small lymphocytic lymphoma with tonsillar enlargement. Exclusion criteria were non-English articles, case reports with incomplete data, and non-CLL patients with tonsillar hypertrophy. Two independent reviewers evaluated the articles for inclusion by looking through the titles and abstracts of the search records. In cases of disagreement, a third reviewer was consulted to reach a consensus.

Results

The search revealed 14 case reports of patients with CLL/SLL and tonsillar enlargement (Table 1). The review revealed that the reported patients ranged in age from 41 to 66 years, with both males and females affected. Most patients presented with obstructive symptoms, such as dysphagia, sore throat, stridor, or OSA. A few were incidental diagnoses or presented with a neck mass. The majority of patients had bilateral tonsillar hypertrophy; a smaller number had unilateral (right or left) tonsil involvement. WBC counts ranged widely (from normal to 116.9 × 10⁹/L), and LDH was usually normal or not reported (NR). 

**Table 1:** Characteristics of patients with chronic lymphocytic leukemia/small lymphocytic lymphoma with tonsillar enlargement CLL: chronic lymphocytic leukemia; OSA: obstructive sleep apnea; NR: not reported; CHOP: cyclophosphamide, hydroxydaunomycin (doxorubicin), oncovin (vincristine), and prednisone M: male; F: female; OSA: obstructive sleep apnea; LBCL: large B cell lymphoma

Reference	Age in years/gender	Presenting Complaint	Tonsillar involvement	Diagnosis/RIA stage	WBC Count (x 10^9^)(normal range: 4-11)	Management	Outcome
[4]	62 F	Globus sensation, dysphagia, dyspnea and stridor	Bilateral hypertrophy, not painful	Known CLL Ria stage II	8	Urgent tracheostomy. Six cycles of BR	Achieved CR
[5]	58 F	Dysphagia, neck pain, fatigue, and shortness of breath (impending airway compromise)	Right tonsil, painful	Concurrent squamous cell carcinoma (SCC) and CLL	83.6	Completed rituximab then started on high-dose cisplatin (25 mg/m2) weekly for 6 cycles and radiotherapy.	Symptomatic improvement. Follow up in different facility.
[6]	66 M	Obstructive sleep apnea	Hypertrophy of the adenoids and lingual tonsils	CLL, no biopsy of the tonsil	Lymphocytosis present	CHOP chemotherapy	Excellent clinical response, resolution of sleep apnea
[7]	54 F	Dysphagia, sore throat, respiratory difficulty	Bilateral hypertrophy	CLL with interfollicular pattern	10.8	Bilateral tonsillectomy	Not reported
[8]	56 M	Incidental	Bilateral hypertrophy	New diagnosis,B-cell CLL	9.9	Incidental diagnosis through pharyngoplasty and tonsillectomy. Observation	Stable disease on follow up
[9]	62 M	Sore throat, dysphagia, airway obstruction	Bilateral hypertrophy	Rapidly progressive CLL, shortly after discontinuation of Ibrutinib maintenance therapy.	116.91	Fiberoptic nasotracheal intubation; Tonsillar biopsy, CHOP chemotherapy	Patient expired
[10]	52 M	Enlarged left cervical lymph node	Enlarged left tonsil.	SLL with SCC	Normal	Bilateral tonsillectomy, biopsies, and selective left neck dissection. Concurrent chemoradiation regimen	Clinical remission
[11]	NR	Obstructive sleep apnea	Bilateral hypertrophy	CLL, new diagnosis	Not reported	Tonsillectomy	Symptomatic improvement, but OSA persisted
[12]	51 M	Obstructive sleep apnea	Bilateral hypertrophy	Primary CLL of the palatine tonsil, new diagnosis	Not reported	Bilateral tonsillectomy	Not reported
[13]	41 M	Severe obstructive sleep apnea	Bilateral hypertrophy	SLL, new diagnosis	Normal	Uvulopalatopharyngoplasty; lymphoma treatment	Sleep apnea improved, clinical remission
[14]	62 M	Bilateral neck fullness	Bilateral hypertrophy	CLL, known	Leukocytosis present	Not reported	Not reported
[15]	54 M	Obstructive sleep apnea	Bilateral hypertrophy	SLL	Not reported	Not reported	Not reported
[16]	63	Enlarged cervical lymph nodes and tonsils	Bilateral tonsillar hypertrophy	Known CLL with new plasmacytoma of the tonsils	Lymphocytosis 8.5	Fludarabine, cyclophosphamide, and rituximab, with no response, 4 cycles of R-CHOP, finally responded to myeloma treatment	Still alive 3 years after treatment without any lymphadenopathy
[17]	65 M	Dysphagia	Left-sided tonsillar enlargement	Known CLL transformed to diffuse LBCL	Absolute lymphocyte count 29.78	Six cycles of R-CHOP	Relapsed after 6 months

Surgical: Bilateral or unilateral tonsillectomy (diagnostic and therapeutic in some). 

Medical: Chemotherapy (CHOP (cyclophosphamide, doxorubicin, vincristine, and prednisone), rituximab + cisplatin), radiotherapy, or observation in stable disease. 

Several cases achieved clinical remission or symptomatic improvement (including resolution of OSA in some). One case had stable disease under observation. One patient with aggressive disease expired despite CHOP therapy. There are two patients with known CLL who developed tonsillar enlargement due to squamous cell carcinoma. Most of the data regarding the cytogenetics and staging were not available. Only one patient transformed to diffuse large B-cell lymphoma, and one patient developed plasmacytoma.

Discussion

Patients with chronic lymphocytic leukemia are often asymptomatic and are mostly diagnosed incidentally based on routine blood tests that show absolute lymphocytosis or rarely present with complications [1,18]. It is also associated with diffuse lymphadenopathy that most commonly affects the cervical, supraclavicular, and axillary areas. Tonsillar involvement is quite rare, with only a handful of cases reported in the literature (see Table 1). Patients may exhibit a diverse range of presentations, from being completely asymptomatic to severe bilateral tonsillar hypertrophy that manifests as obstructive symptoms such as dysphagia, apnea, and even respiratory distress and airway compression. Unfortunately, given the rarity of this presentation of CLL, it is often mistaken for either infectious tonsillitis or benign hyperplasia, where patients are treated symptomatically, thus leading to delayed diagnosis. This may explain why patients often present with obstructive symptoms such as dysphagia or sleep apnea, or in advanced cases, with airway compromise. Therefore, a lack of response to supportive treatment should raise suspicion of an underlying serious condition that requires further investigation. Clinically, it isn't easy to differentiate CLL tonsillar hypertrophy from other causes. From the aforementioned reported cases, it is clear that CLL-related tonsillar hypertrophy is usually bilateral but can also be unilateral. Moreover, tonsillar hypertrophy can occur in conjunction with lymphocytosis and a normal white blood cell count, which makes clinical assessment alone insufficient to differentiate CLL from non-CLL tonsillar hypertrophy.

Diagnosis of CLL is based on two criteria: the presence of sustained lymphocytosis (≥ 5,000 cells/mm³) for three months, in addition to the presence of monoclonal B lymphocytes confirmed by flow cytometry with expression of B cell-associated antigens (CD19, CD20 (typically dim), and CD23) and expression of CD5 [19]. Lymph node biopsy or bone marrow aspiration is not often needed to confirm the diagnosis. However, it is worth noting that, for CLL patients with tonsillar enlargement, diagnosis was primarily made through tonsillectomy tissue biopsy, as white blood cell counts were sometimes normal, especially in cases presenting with mild symptoms. On the other hand, patients presenting with severe obstructive symptoms had severe leukocytosis that already raised the suspicion for an underlying malignant pathology, which was later confirmed by the CLL immunophenotype. Moreover, tissue biopsy can provide insight into the presence of concurrent malignancies that often affect the tonsils, such as squamous cell carcinoma, as seen in the case [5,10].

Interestingly, there are few reported cases of tonsillar enlargement associated with CLL, despite the tonsils being part of the reticuloendothelial system. Tonsillar enlargement can occur during the disease and serve as a presentation of CLL. The effect of tonsillar enlargement on CLL outcomes is unclear. Possible causes of tonsillar enlargement in CLL include lymphoid tissue involvement of the tonsils, as is the case in most reported cases. Interestingly, only one reported case showed that tonsillar involvement is due to CLL transformation into diffuse large B-cell lymphoma. Other causes include secondary malignancy, as reported in two patients with squamous cell carcinoma and one patient with plasmacytoma [5,10,16]. These cases support the approach of confirming tonsillar enlargement with biopsy/tonsiliectomy because these cases would be missed if it were assumed to be related to CLL.

Typically, CLL follows a benign course; the indications for treating CLL include progressive marrow failure, which may manifest as anemia or thrombocytopenia, autoimmune anemia or thrombocytopenia that is not responsive to steroids, massive splenomegaly, or lymphadenopathy [19]. Additionally, if there is symptomatic or functional extranodal involvement [19], the latter indication may be suitable for symptomatic tonsillar enlargement, as seen in our reported cases. The management strategy for tonsillar CLL varies widely depending on the severity of symptoms. For example, in asymptomatic patients with normal counts diagnosed incidentally, a “watch and wait” approach can be adopted. However, in patients with obstructive symptoms, tonsillectomy is crucial not only for diagnosis but also for symptomatic relief. In patients with severe airway compromise and rapid progression, nasotracheal intubation or tracheostomy is life-saving. Further management with systemic therapy is often needed, especially in advanced cases. Fortunately, several cases have been noted to achieve symptomatic resolution and clinical remission of the disease through various treatment approaches, and only one patient has expired, as reported in Mangin et al. [9].

The major limitation of this review is the small number of cases, given the rarity of tonsillar enlargement in CLL. Consequently, conclusions regarding the prognosis, outcome, and treatment of CLL tonsillar involvement remain individualized. Other possible limitations include reporting bias. Tonsillar involvement appears to be low, which can be due to under-reporting or reporting bias, where only tonsillar enlargement that leads to airway compromise or obstructive sleep apnea is reported. Another limitation is the English language, as only English literature was searched. Tonsillar involvement might be reported in other studies. Additionally, it appears that not all authors were eager to report the workup; most cases lack reports on flow cytometry and fluorescent in situ hybridization (FISH) studies.

## Conclusions

Tonsillar hypertrophy in CLL represents a rare but important diagnostic and therapeutic consideration. Clinicians should maintain a high index of suspicion for malignant infiltration in patients with atypical or refractory tonsillar enlargement, as it is challenging to identify CLL-related tonsillar enlargement solely based on clinical findings. In patients with CLL who develop new or asymmetric tonsillar enlargement, the lesion should not be automatically attributed to CLL involvement. A biopsy is mandatory to exclude a second primary malignancy (e.g., squamous cell carcinoma) or CLL transformation. Tonsillectomy will be both diagnostic and therapeutic, relieving obstructive symptoms. Early diagnosis facilitates appropriate management, prevents airway compromise, and enables timely initiation of systemic therapy when indicated.
